# The Integrity of the Cell Wall and Its Remodeling during Heterocyst Differentiation Are Regulated by Phylogenetically Conserved Small RNA Yfr1 in *Nostoc* sp. Strain PCC 7120

**DOI:** 10.1128/mBio.02599-19

**Published:** 2020-01-21

**Authors:** Manuel Brenes-Álvarez, Agustín Vioque, Alicia M. Muro-Pastor

**Affiliations:** a Instituto de Bioquímica Vegetal y Fotosíntesis, Consejo Superior de Investigaciones Científicas and Universidad de Sevilla, Seville, Spain; Université Paris Diderot, Paris, France; University of Würzburg

**Keywords:** *Anabaena*, cyanobacteria, heterocyst differentiation, regulatory RNAs, small RNAs

## Abstract

Yfr1 is a strictly conserved small RNA in cyanobacteria. A bioinformatic prediction to identify possible interactions of Yfr1 with mRNAs was carried out by using the sequences of Yfr1 from several heterocyst-forming strains, including *Nostoc* sp. strain PCC 7120. The results of the prediction were enriched in genes encoding outer membrane proteins and enzymes related to peptidoglycan biosynthesis and turnover. Heterologous expression assays with Escherichia coli demonstrated direct interactions of Yfr1 with mRNAs of 11 of the candidate genes. The expression of 10 of them (*alr2458*, *alr4550*, *murC*, *all4829*, *all2158*, *mraY*, *alr2269*, *alr0834*, *conR*, *patN*) was repressed by interaction with Yfr1, whereas the expression of *amiC2*, encoding an amidase, was increased. The interactions between Yfr1 and the 11 mRNAs were confirmed by site-directed mutagenesis of Yfr1. Furthermore, a *Nostoc* strain with reduced levels of Yfr1 had larger amounts of *mraY* and *murC* mRNAs, supporting a role for Yfr1 in the regulation of those genes. *Nostoc* strains with either reduced or increased expression of Yfr1 showed anomalies in cell wall completion and were more sensitive to vancomycin than the wild-type strain. Furthermore, growth in the absence of combined nitrogen, which involves the differentiation of heterocysts, was compromised in the strain overexpressing Yfr1, and filaments were broken at the connections between vegetative cells and heterocysts. These results indicate that Yfr1 is an important regulator of cell wall homeostasis and correct cell wall remodeling during heterocyst differentiation.

## Abstract (precis)

Bacterial small RNAs (sRNAs) are important players affecting the regulation of essentially every aspect of bacterial physiology. The cell wall is a highly dynamic structure that protects bacteria from their fluctuating environment. Cell envelope remodeling is particularly critical for bacteria that undergo differentiation processes, such as spore formation or differentiation of heterocysts. Heterocyst development involves the deposition of additional layers of glycolipids and polysaccharides outside the outer membrane. Here, we show that a cyanobacterial phylogenetically conserved small regulatory RNA, Yfr1, coordinates the expression of proteins involved in cell wall-related processes, including peptidoglycan metabolism and transport of different molecules, as well as expression of several proteins involved in heterocyst differentiation.

## INTRODUCTION

Bacterial cell envelopes are multilayered structures that delimit the interior of the cell from its environment. The essential component of bacterial cell walls is peptidoglycan, a strong and flexible mesh that protects the cell against osmotic pressure and contributes to the shape of the cell. According to the architecture of the envelopes, bacteria are classified into two groups. Gram-positive strains have a thick peptidoglycan layer (30 to 100 nm) surrounding the cytoplasmic membrane, while Gram-negative strains have a thin peptidoglycan layer (only a few nanometers) between the inner, cytoplasmic membrane and a second, outer membrane (1). Cyanobacteria are Gram-negative. However, the peptidoglycan layer between the inner and outer membranes is relatively thick (15 to 30 nm in filamentous strains) and characterized by extensive cross-linking, rather resembling the architecture of Gram-positive bacteria (2).

The sacculus of peptidoglycan is a dynamic structure that must adapt to the growth of the cells, the separation of daughter cells during cell division, the turnover of peptidoglycan, or the assembly of large transenvelope complexes (e.g., secretion systems). In addition, cellular differentiation processes that affect cell envelopes involve peptidoglycan remodeling, performed by different murein hydrolases, such as *N*-acetylmuramoyl-l-Ala amidases (Ami enzymes) (reviewed in reference 3).

*Nostoc* sp. strain PCC 7120 is a filamentous cyanobacterium that under nitrogen deprivation differentiates heterocysts (specialized cells devoted to N_2_ fixation) in a semiregular pattern (4). Heterocyst differentiation involves biochemical and morphological changes that provide a micro-oxic environment for nitrogenase, an O_2_-labile enzyme. In addition to inactivating O_2_-producing photosystem II and increasing the O_2_ consumption rate, heterocysts have a special cellular envelope with two extra layers, an external one composed of polysaccharides (heterocyst envelope polysaccharides [HEPs]) and a laminated, internal layer composed of glycolipids (heterocyst glycolipids [HGLs]) that acts as a barrier to gas diffusion (5). Both heterocyst-specific layers are deposited outside the outer membranes of these cells.

Under nitrogen-fixing conditions, there is a metabolic division of labor between vegetative cells and heterocysts in cyanobacterial filaments. Heterocysts feed vegetative cells with fixed nitrogen and obtain fixed carbon in return. The growth of nitrogen-fixing filaments depends on the transport and exchange of metabolites between vegetative cells and heterocysts (4). One route for this exchange of metabolites may consist of diffusion from cytoplasm to cytoplasm through septal junctions, protein structures that allow intercommunication between the cytoplasms of adjacent cells (6–9). These structures traverse the septal peptidoglycan disks through perforations called “nanopores” (7), which may be made by murein hydrolases.

Mutants with mutations in the murein amidases AmiC1 (*alr0092*) (10) and AmiC2 (*alr0093*, *hcwA*) (11, 12) show a significant reduction in the number of nanopores and defects in diazotrophic growth. In addition, strains with mutations in the peptidoglycan synthesis enzymes MurC (*alr5065*) and MurB (*alr5066*) (13) and in some penicillin binding proteins (PBPs) (those encoded by *all2981*, *alr4579*, and *alr5051*) (14, 15) show alterations in heterocyst differentiation and growth defects in media without combined nitrogen. All this evidence points to the synthesis and remodeling of peptidoglycan being essential for proper heterocyst differentiation and diazotrophic growth. In fact, several genes related to peptidoglycan metabolism are transcribed from HetR-dependent, heterocyst-specific promoters (16, 17).

The maintenance and assembly of the outer membrane rely on proteins of the Omp85 family. Omp85 is essential for outer membrane maturation in Escherichia coli (18), and an *alr2269* mutant (encoding an Omp85 homolog in *Nostoc* sp. PCC 7120) showed a disturbed outer membrane and, as a consequence, higher sensitivity to harmful substances, such as erythromycin (19). Molecules enter the periplasm through different transporters, such as porin-like proteins and TonB-dependent transporters. Porins usually allow the diffusion of hydrophilic molecules of a size up to 600 Da with low selectivity (20). The genome of *Nostoc* sp. PCC 7120 encodes a general porin (Alr0834), OprB-like porins (Alr4550 and All4499) (21), and several TonB-dependent transporters (All2158, All3310, All4026) (22).

Yfr1 is one of the first small RNAs (sRNAs) identified in cyanobacteria and was initially described in unicellular picocyanobacteria (23). Bioinformatic prediction and the use of a heterologous reporter system in E. coli demonstrated that Yfr1 can interact with the mRNAs encoding two porins in *Prochlorococcus* MED4 (24). Recently, a global approach allowed the identification of the targetome of Yfr1 from *Prochlorococcus* MED4 (25). In Synechococcus elongatus PCC 6301, Yfr1 is highly expressed, with slight abundance changes in cells exposed to high-salt stress or oxidative stress. A Yfr1 mutant showed reduced growth under iron limitation, high-salt stress, or oxidative stress (26).

Yfr1 has been identified in all cyanobacterial genomes analyzed, from the minimal genomes of unicellular strains of the *Prochlorococcus-Synechococcus* lineages to much larger genomes of complex filamentous strains, such as *Nostoc*, able to undergo cellular differentiation processes (27). This broad occurrence suggests a widely conserved function for Yfr1. Interestingly, a fully conserved sequence motif is present in all Yfr1 homologs across the different cyanobacterial clades (27, 28). We have identified and verified the interaction of Yfr1 with several mRNAs whose products are involved in peptidoglycan metabolism and envelope biogenesis and maintenance in *Nostoc* sp. PCC 7120, including several proteins required for proper differentiation of functional heterocysts and therefore N_2_ fixation. Our results suggest that Yfr1 may regulate the composition and remodeling of envelopes of heterocyst-forming cyanobacteria.

## RESULTS

### Validation of putative Yfr1 targets in E. coli.

According to previous studies, Yfr1 is expressed constitutively, with only slight expression changes under different growth conditions in the unicellular cyanobacteria Synechococcus elongatus PCC 6301 (26) and *Prochlorococcus* MED4 (25). We tested the expression of Yfr1 in *Nostoc* sp. PCC 7120 (see Fig. S1 in the supplemental material). As with the observations made in the case of unicellular strains, Yfr1 had a relatively strong expression and its levels changed only slightly due to nitrogen deficiency or high-light stress, two conditions that lead to pronounced changes in cyanobacterial gene expression. As previously shown, Yfr1 accumulates in the form of two transcripts with slightly different sizes (28).

10.1128/mBio.02599-19.1 FIG S1 Expression of Yfr1 in *Nostoc* sp. PCC 7120. (A and B) Northern blot analyses with total RNA extracted at different times after removal of combined nitrogen (A) and with total RNA from cells growing in the presence of ammonia at 50 μE m^−2^ s^−1^ and incubated at 500 μE m^−2^ s^−1^ (high-light stress [HL]) for the indicated times (B). All membranes were hybridized with probes for Yfr1 and 5S RNA as a loading control. Download FIG S1, PDF file, 0.4 MB. Copyright © 2020 Brenes-Álvarez et al. 2020 Brenes-Álvarez et al. This content is distributed under the terms of the Creative Commons Attribution 4.0 International license.

**FIG 1:**
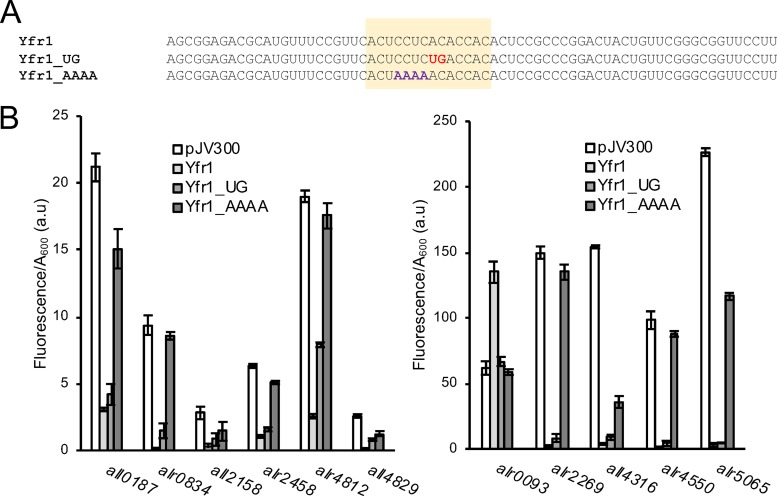
Verification of the interaction between Yfr1 and several mRNAs using an *in vivo* reporter system in E. coli. (A) Different versions of Yfr1 used to validate Yfr1’s interaction with several targets. The top line is the wild-type sequence. Mutations introduced into Yfr1 at positions 31 and 32 (AC to UG, Yfr1_UG) and 27 to 30 (CCUC to AAAA, Yfr1_AAAA) are shown in red and purple, respectively. The fully conserved motif of Yfr1 is shaded in orange. (B) Fluorescence of E. coli cultures bearing different combinations of plasmids expressing wild-type or mutated versions of Yfr1 or a control RNA (pJV300) and different 5′-UTRs fused to *sfgfp*. The data are presented as the means and standard deviations of the results from 8 independent colonies after subtraction of fluorescence in cells bearing pXG0 and normalized for cell density (*A*_600_). Results are presented in two graphs according to the different scales required. a.u., arbitrary units.

The sequence of Yfr1 from *Nostoc* sp. PCC 7120 is shown in Fig. 1A. Because prediction of sRNA targets can be improved when comparative phylogenetic information is taken into account (29), we used CopraRNA, a software to predict potential interactions between sRNAs and mRNA targets that are conserved among a set of organisms (29, 30). Using the sequences of Yfr1 homologs from 10 different heterocystous strains (see Materials and Methods for details), the resulting list of predicted targets (Table S1) showed a significant enrichment in transporters, enzymes related to cell wall synthesis or remodeling, and proteins located in the outer membrane. Among the predicted targets were several mRNAs encoding enzymes involved in peptidoglycan metabolism, such as *alr2458* (alanine racemase), *alr5065* (*murC*), *all4316* (*mraY*), *alr0093* (*hcwA*, *amiC2*), or *all3826* (a penicillin binding protein [PBP]). Several of the predicted targets corresponded to proteins known to be located in the outer membrane (21), including *alr0834*, encoding a homolog of the two previously validated targets of Yfr1 in *Prochlorococcus* (24), *alr4550* and *all4499* (OprB-like porins), *all2158* and *all4026* (TonB-dependent transporters), proteins related to the biogenesis of outer membrane (*alr2269*, Omp85), or a component of a TolC-like secretion system (*alr2887*). In addition, the proteins encoded by *all0089* and *all3310*, also predicted targets, have previously been described as being located in the outer membrane, although their possible function is unknown (31). Among the top 50 predicted targets were also two genes (*patN* and *conR*) which are related to cell wall maintenance and also involved in certain aspects of heterocyst differentiation. Biased inheritance of PatN, located in the cytoplasmic membrane, has been related to the differentiation of certain cells into heterocysts (32), whereas ConR is essential for proper septum formation between cells (33).

10.1128/mBio.02599-19.5 TABLE S1 Top 50 predicted targets of Yfr1. Download Table S1, DOCX file, 0.02 MB. Copyright © 2020 Brenes-Álvarez et al. 2020 Brenes-Álvarez et al. This content is distributed under the terms of the Creative Commons Attribution 4.0 International license.

We have verified the interaction between Yfr1 and 11 selected targets (shown in gray in Table S1), using a heterologous reporter system (34) in which the 5′ untranslated region (5′-UTR) of the predicted target mRNAs (plus sequences encoding the first 10 to 20 amino acids of the corresponding protein) is translationally fused to the gene *sfgfp* and coexpressed in E. coli with Yfr1 or with a control, unrelated RNA. We were able to measure the fluorescence of cells carrying fusions to superfolder green fluorescent protein (sfGFP) of every target, indicating that the translation initiation regions of these mRNAs were functional in E. coli. In all cases analyzed, except for the *alr0093*::*sfgfp* strain, the fluorescence of cells carrying *sfgfp* fusions significantly decreased when Yfr1 was coexpressed, indicating a negative effect of Yfr1 on the expression of sfGFP (Fig. 1B). In contrast, in the case of the *alr0093*::*sfgfp* strain, the fluorescence was higher in the presence of Yfr1.

The interactions between Yfr1 and the 5′-UTRs of the 11 target mRNAs analyzed, as predicted by IntaRNA (35), are shown in Fig. S2. In all cases, the predicted interaction involves the conserved region of Yfr1 and takes place in a region of the mRNA located just upstream of the translational start site of the corresponding gene, except in *alr2269* and *alr0093*, where the interaction is predicted further upstream. *alr0093* is the only fusion whose expression was activated by interaction with Yfr1 (Fig. 1B).

10.1128/mBio.02599-19.2 FIG S2 Interactions predicted by IntaRNA between Yfr1 and several mRNAs. Nucleotides of 5′-UTRs are numbered with respect to the start of the coding sequence (start codons are indicated in bold and underlined). Positions of the mutations introduced in Yfr1_UG are shown in red. Positions of the mutations introduced in Yfr1_AAAA are shown in purple. Download FIG S2, PDF file, 0.1 MB. Copyright © 2020 Brenes-Álvarez et al. 2020 Brenes-Álvarez et al. This content is distributed under the terms of the Creative Commons Attribution 4.0 International license.

To verify that the interactions with the predicted targets involved the conserved region of Yfr1, we designed two mutated versions of Yfr1, one altered at positions 31 and 32 (AC to UG, Yfr1_UG) and another one altered at positions 27 to 30 (CCUC to AAAA, Yfr1_AAAA) (Fig. 1A). In all cases, Yfr1_UG had a slightly weaker effect than wild-type Yfr1 in the level of the GFP fluorescence of strains bearing the sfGFP fusions. In contrast, mutations introduced in Yfr1_AAAA strongly affected the magnitude of the change in GFP fluorescence of the fusion proteins, compared with the change due to coexpression with wild-type Yfr1 (Fig. 1B). These results strongly suggest that the highly conserved region of Yfr1 was involved in the interaction between Yfr1 and its targets, as previously reported for Yfr1 in *Prochlorococcus* (24).

**FIG 2:**
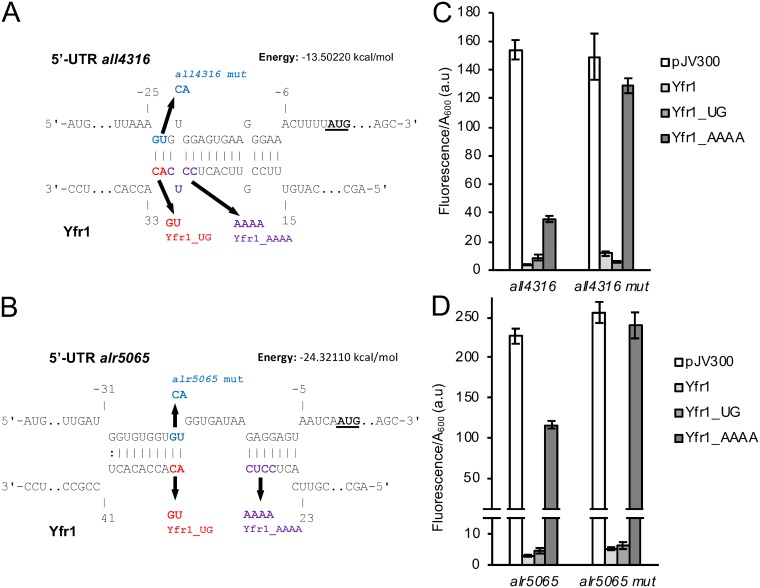
Interaction of Yfr1 and the 5′-UTRs of *all4316* and *alr5065* in E. coli. (A and B) Interactions predicted by IntaRNA between Yfr1 and the 5′-UTRs of *all4316* (A) and *alr5065* (B). Nucleotides of the 5′-UTRs are numbered with respect to the start of the coding sequence (start codons are indicated in bold and underlined). Mutations introduced into Yfr1 at positions 31 and 32 (AC to UG, Yfr1_UG) and compensatory mutations introduced into the 5′-UTRs of the mRNAs are shown in red and blue, respectively. The additional mutations introduced into Yfr1 at positions 27 to 30 (CCUC to AAAA, Yfr1_AAAA) are shown in purple. (C and D) Fluorescence of E. coli cells bearing combinations of plasmids expressing different versions of Yfr1 and the wild-type or mutated versions of the 5′-UTRs of *all4316* (C) and *alr5065* (D). The data are presented as means and standard deviations of the results from 8 independent colonies after subtraction of fluorescence in cells bearing pXG0 and normalized for cell density (*A*_600_).

We further analyzed the interaction between Yfr1 and the 5′-UTRs of two genes involved in peptidoglycan synthesis, *all4316* (*mraY*) and *alr5065* (*murC*). We designed compensatory mutations in the 5′-UTRs of the two genes that would restore the interaction with Yfr1_UG (Fig. 2A and B) and weaken the interaction with Yfr1_AAAA. When Yfr1_UG was combined with the mutated version of the 5′-UTR of *all4316* (*all4316* mut), a slightly stronger reduction of fluorescence with respect to that of cells coexpressing the unrelated control RNA was observed with Yfr1_UG than with wild-type Yfr1. In the case of Yfr1_AAAA, a much weaker reduction in fluorescence was observed when it was combined with *all4316* mut than when it was combined with the wild-type version of the 5′-UTR of *all4316* (Fig. 2C). In the case of *all5065*, no significant differences were observed when Yfr1_UG was combined with the wild-type or mutant versions of the 5′-UTR, but again Yfr1_AAAA produced a much weaker reduction in fluorescence when it was combined with *all5065* mut than when it was combined with wild-type *all5065* (Fig. 2D). All together, these results support the interaction of Yfr1 with the 5′-UTRs of both genes.

### Yfr1 affects *all4316* (*mraY*) and *alr5065* (*murC*) expression in *Nostoc* sp. PCC 7120.

**FIG 3:**
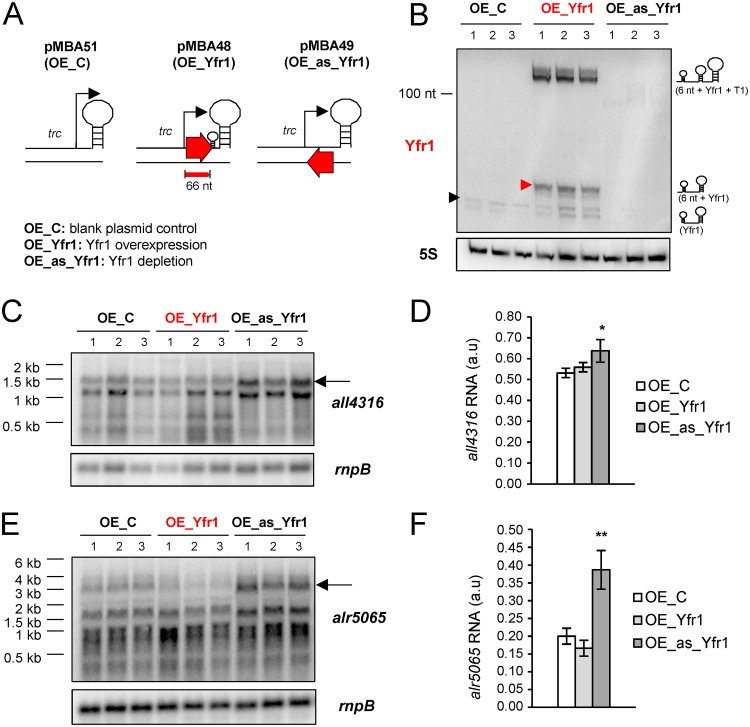
Expression of Yfr1, *all4316*, and *alr5065* in OE_C, OE_Yfr1, and OE_as_Yfr1 *Nostoc* strains. (A) Schemes of plasmids pMBA51 (OE_C), pMBA48 (OE_Yfr1), and pMBA49 (OE_as_Yfr1). The transcription start site (bent arrows), T1 terminator (large stem loops), Rho-independent terminator of Yfr1 (small stem loops), *trc* promoter, probe for Yfr1 (red thick line), and sequences corresponding to Yfr1 (red arrows) are indicated. nt, nucleotides. (B, C, E) Northern blots with RNAs from three independent clones of the OE_C, OE_Yfr1, and OE_as_Yfr1 strains grown in the presence of ammonia and hybridized with probes for Yfr1 (B), *all4316* (C), *alr5065* (E), and 5S RNA (B) or *rnpB* (C, E) as loading controls. Endogenous Yfr1 (black triangle) and Yfr1 expressed constitutively from the *trc* promoter (red triangle) are indicated. (D, F) Quantification of *all4316* (D) or *alr5065* (F) expression. The bands corresponding to full-length mRNA (indicated by an arrow in panels C and E) were used for quantification. Data are presented as the means ± standard deviations of the signals in the largest intact band normalized to the *rnpB* signal (three individual clones of each strain). *, *P* < 0.05; **, *P* < 0.01 (*t* test).

In order to study the function of Yfr1 in *Nostoc*, we prepared strains with altered levels of Yfr1 (Table S2). Because Yfr1 accumulates at relatively high levels in the wild-type strain, to overexpress Yfr1, we introduced in *Nostoc* a plasmid designed for very strong expression from the *trc* promoter (strain OE_Yfr1) (Fig. 3A). In this plasmid, the segment cloned downstream of the *trc* promoter is transcribed to the T1 transcriptional terminator from the *rrnB* gene of E. coli (36). A 6-nucleotide tag was introduced between the transcriptional start site of the *trc* promoter and the DNA segment encoding Yfr1 so that the native endogenous molecules of Yfr1 could be distinguished from Yfr1 molecules expressed from the *trc* promoter based on their lengths. In order to reduce the amount of Yfr1 without altering the expression of the downstream *trxA* gene, we followed a strategy used previously (37) and transformed *Nostoc* with a plasmid bearing the Yfr1 sequence in reverse orientation downstream from the *trc* promoter, so that its transcription generates a sequence perfectly antisense to that of Yfr1 (Fig. 3A), which acts as a sponge, neutralizing Yfr1 (strain OE_as_Yfr1). As a control, we used a *Nostoc* strain with a plasmid without an insert between the *trc* promoter and the terminator (OE_C).

10.1128/mBio.02599-19.6 TABLE S2 Strains. Download Table S2, DOCX file, 0.02 MB. Copyright © 2020 Brenes-Álvarez et al. 2020 Brenes-Álvarez et al. This content is distributed under the terms of the Creative Commons Attribution 4.0 International license.

We analyzed the accumulation of Yfr1 in *Nostoc* strains bearing the above-described constructs by Northern blot hybridization, using three independent clones of the OE_C, OE_Yfr1, and OE_as_Yfr1 strains grown in the presence of combined nitrogen (ammonium). Expression of Yfr1 under the *trc* promoter clearly exceeded endogenous Yfr1 expression, whereas transcription of the sequence antisense to Yfr1 led to complete depletion of endogenous Yfr1 (Fig. 3B). In the OE_Yfr1 strain, termination of the strong transcription from the *trc* promoter at the Rho-independent terminator of Yfr1 was only partial, and most transcripts were terminated at the T1 transcriptional terminator (56 nucleotides downstream) and appeared as longer molecules in Northern blots (Fig. 3B).

We then tested the accumulation of the mRNAs of *all4316* and *alr5065* in the OE-C (control), OE_Yfr1, and OE_as_Yfr1 strains. The accumulation of intact *all4316* and *alr5065* mRNAs was significantly stronger in the OE_as_Yfr1 strain (depleted of Yfr1) than in the OE_C strain (Fig. 3C to F). There was also a slightly reduced accumulation of intact *alr5065* mRNA in the OE_Yfr1 strain with respect to that of the control strain (Fig. 3E and F), and degradation products of *all4316* were clearly observed in strain OE_Yfr1. Taken together, these results indicated that Yfr1 negatively affects the accumulation of *all4316* and *alr5065* mRNAs in *Nostoc* sp. PCC 7120. This is the expected result if inhibition of their translation by Yfr1 results in indirect destabilization of the mRNAs, in agreement with the results obtained with E. coli (Fig. 1 and Fig. S3).

10.1128/mBio.02599-19.3 FIG S3 Comparison of the effects of Yfr1 on the expression of *all4316* and *alr5065* at the mRNA (A, B) and protein (C) levels in E. coli. (A) Northern blot with RNA isolated from E. coli cells bearing plasmid pMBA4 (*all4316-sfgfp*) or pMBA7 (*alr5065-sfgfp*) and a plasmid expressing Yfr1 (pMBA1) or a control RNA (pJV300) hybridized with probes for *all4316*, *alr5065*, Yfr1, and 5S RNA (as a loading control). (B) Quantification of signals in Northern blots. Data are presented as the means ± standard deviations of the signals normalized to the 5S signal (three individual experiments). (C) Fluorescence of the E. coli cultures analyzed in panels A and B. The data are presented as means ± standard deviations of the results from three cultures after subtraction of the fluorescence in cells bearing pXG0 and normalized for cell density (*A*_600_). *, *P* < 0.05; **, *P* < 0.0001 (*t* test). Download FIG S3, PDF file, 1.3 MB. Copyright © 2020 Brenes-Álvarez et al. 2020 Brenes-Álvarez et al. This content is distributed under the terms of the Creative Commons Attribution 4.0 International license.

### Strains with altered levels of Yfr1 show altered cell wall integrity and peptidoglycan synthesis.

**FIG 4:**
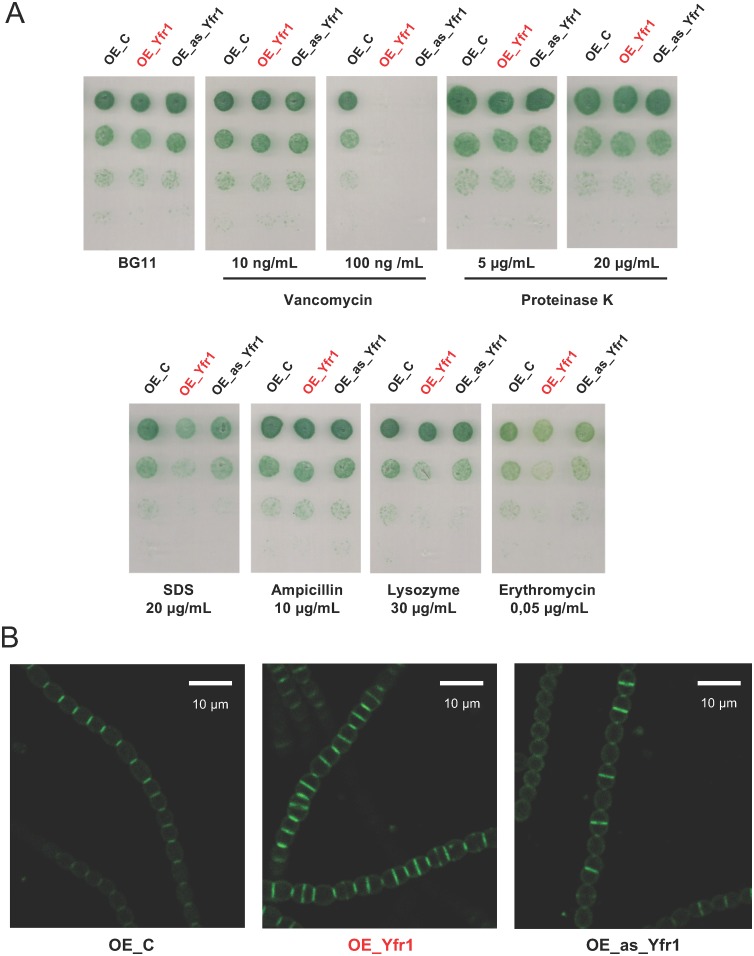
Functionality of the cell wall in strains with altered levels of Yfr1. (A) Growth of OE_C, OE_Yfr1, and OE_as_Yfr1 strains in media containing nitrate (BG11) and supplemented with the indicated substances. Pictures were taken after 10 days of incubation at 30°C. (B) Fluorescence microscopy images of Van-FL-stained filaments of OE_C (left), OE_Yfr1 (center), and OE_as_Yfr1 (right) strains.

We have validated the interaction between Yfr1 and the 5′-UTRs of several mRNAs encoding proteins related to the cell wall (transporters and proteins located in the outer membrane) and peptidoglycan biosynthesis or remodeling. In order to verify the physiological relevance of Yfr1, we tested the effects of several harmful compounds that may affect the growth of strains with a compromised cell wall for the above-described strains with altered levels of Yfr1. Unlike with the OE-C strain, neither the OE_Yfr1 nor the OE_as_Yfr1 (Yfr1-depleted) strains were able to grow on plates containing 100 ng/ml vancomycin, an antibiotic that binds to the nascent peptidoglycan chains (Fig. 4A). In addition, OE_Yfr1 grew slightly worse than the control strain in plates containing SDS or erythromycin (Fig. 4A).

We also visualized the septa between cells by incorporation of a fluorescent derivative of vancomycin (Van-FL) that binds to nascent peptidoglycan chains (38). In comparison to the control strain OE-C, which showed fluorescent septa only between individual cells, OE_Yfr1 showed fluorescent septa in the middle of cells that had not completed division (Fig. 4B). Most of the septa in the OE-Yfr1 strain were wider than those in the OE_C strain, suggesting that cell division was not properly completed. In contrast, strain OE_as_Yfr1 showed very narrow septa between cells that had completed division, suggesting a faster completion of the septa.

### Yfr1 may affect heterocyst differentiation.

**FIG 5:**
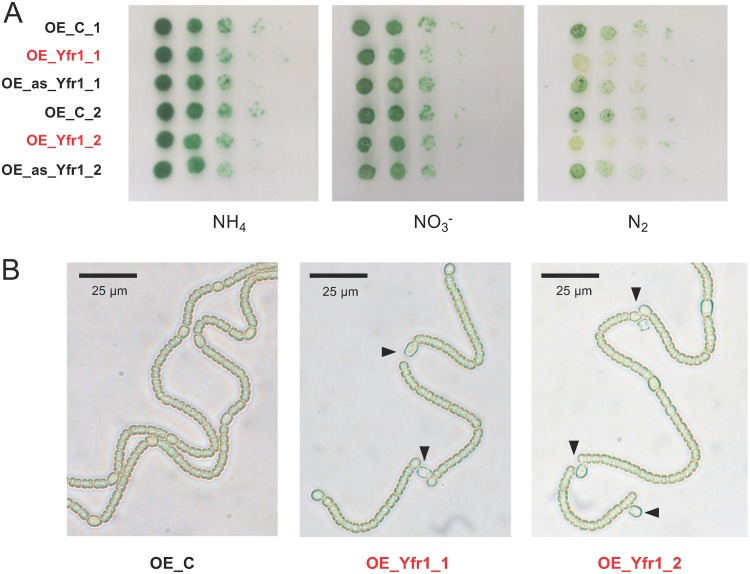
Growth of strains with altered levels of Yfr1. (A) Growth of OE_C, OE_Yfr1, and OE_as_Yfr1 strains in media lacking nitrogen (N_2_) or containing nitrate (NO_3_^–^) or ammonium (NH_4_^+^). Pictures were taken after 10 days of incubation at 30°C. (B) Bright-field images of filaments from the OE_C strain and from two independent clones of strain OE_Yfr1 streaked on top of BG11_0_ plates. The broken connections between heterocysts and vegetative cells are indicated with black triangles.

The differentiation of heterocysts involves important morphological changes, including the secretion of specific components of envelopes (HEPs and HGLs) outside the outer membrane. Peptidoglycan remodeling seems essential for the correct deposition of these envelopes as well as for the proper communication between vegetative cells and heterocysts (10–12, 14, 15). In order to analyze completion of heterocyst differentiation in strains OE_Yfr1 and OE_as_Yfr1, we tested their growth in plates containing different nitrogen sources (Fig. 5A). Whereas strain OE_as_Yfr1 showed no difference from the OE_C strain, strain OE_Yfr1 grew worse than the control strain in plates without combined nitrogen, a nutritional condition that requires differentiation of functional nitrogen-fixing heterocysts. Strain OE_Yfr1 was unable to grow in liquid media without combined nitrogen (data not shown). The visualization of filaments of strain OE_Yfr1 streaked on top of plates of media lacking combined nitrogen showed a patterned differentiation of heterocysts, but the filaments appeared broken between heterocysts and adjacent vegetative cells (Fig. 5B).

## DISCUSSION

Yfr1 is an sRNA conserved in all cyanobacterial genomes (27, 28). The conservation of an sRNA in organisms with such a wide variety of morphologies, ecological niches, and developmental processes may suggest a regulatory function of a general aspect of the physiology of cyanobacteria. The prediction of targets by CopraRNA (29, 30) for Yfr1 homologs in heterocyst-forming cyanobacteria showed a remarkable enrichment in transporters and proteins located in the outer membrane as well as proteins involved in the synthesis or remodeling of peptidoglycan (see Table S1 in the supplemental material). Target prediction was performed using genomes of heterocystous strains, and in fact, among the top 50 predicted targets, there are some genes of unknown function, such as *alr4714*, *asl4743*, *all0997*, and *alr0255*, that appear conserved only in this cyanobacterial clade (39).

Using a heterologous assay with E. coli, we have demonstrated the effect of Yfr1 on the expression of 11 selected predicted targets and have verified that Yfr1 mostly downregulates the expression of the targets (10 cases), but it can also exert a positive regulation, as in the case of *alr0093* (Fig. 1B). We could demonstrate that regulation was exerted via base pairing of the highly conserved motif of Yfr1 with the mRNAs (Fig. 1 and 2). Most of the predicted interaction sites were located in the translation initiation region (Fig. S2). However, the interaction between Yfr1 and *alr0093* was located far upstream from the start codon (Fig. S2), opening the possibility that the positive regulation of this particular mRNA is operated through a conformational change in its 5′-UTR that might improve translation. Using compensatory mutations, we have mapped the interactions between Yfr1 and the 5′-UTRs of *all4316* and *alr5065* to the ribosome binding region (Fig. 2). These interactions are consistent with a negative regulation based on interference with ribosome access. In fact, the reduction in the corresponding RNA levels is only around 30 to 50% (Fig. S3A and B), whereas the fluorescence of translational reporters fused to the 5′-UTRs of *all4316* and *alr5065* is reduced more than 90% in the presence of Yfr1 (Fig. S3C), further suggesting a mechanism involving translational interference rather than alteration of mRNA stability.

In order to assess the effects of Yfr1 in *Nostoc*, we overexpressed Yfr1 and anti-Yfr1 RNA under the strong and constitutive *trc* promoter (Fig. 3). Indeed, by analyzing *Nostoc* strains with altered levels of Yfr1, we observed a higher accumulation of full-length mRNAs for *all4316* and *alr5065* in strain OE_as_Yfr1 (Fig. 3C to F), which is depleted of Yfr1 (Fig. 3B), than in the control OE-C strain. This result was consistent with the negative regulation exerted by Yfr1 on these targets, as validated in the E. coli system (Fig. 2).

Vancomycin binds to the d-alanyl–d-alanine terminus of nascent glycan chains, preventing the cross-linking between two glycan strands (40). Strains OE_Yfr1 and OE_as_Yfr1 were more sensitive to vancomycin than strain OE_C strain (Fig. 4A), suggesting that both strains have alterations in peptidoglycan integrity, consistent with altered amounts of *murC* and *mraY* mRNAs. In addition, the poor growth of the OE_Yfr1 strain in plates containing SDS (a detergent) or erythromycin (a macrolide antibiotic) suggested additional defects in the integrity and permeability of the envelopes of this strain. This result is consistent with negative regulation of Yfr1 on *alr2269* mRNA (Omp85), since an *alr2269* mutant showed greater sensitivity to SDS and erythromycin (19). When we measured the incorporation of Van-FL, a fluorescent derivative of vancomycin, we observed opposite phenotypes for the OE_Yfr1 and OE_as_Yfr1 strains (Fig. 4B). OE_Yfr1 had wider, frequently nascent septa in the middle of cells that had not finished their previous division, whereas OE_as_Yfr1 had narrower septa, and only some new septa were starting their completion. These results might be consistent with altered peptidoglycan synthesis and remodeling mediated by a negative regulation by Yfr1 of *all4316* (*mraY*) and *alr5065* (*murC*) and a positive regulation of *alr0093* (*amiC2*). Modulation of the amounts of the corresponding enzymes may facilitate a faster completion of the peptidoglycan layer in OE_as_Yfr1 and a slower completion of the peptidoglycan layer in OE_Yfr1, although these strains may differ also in other aspects of cell division. In fact, ConR, a protein of the LytR-CpsA-Psr superfamily involved in septum formation (33), is one of the targets of Yfr1 verified in this work (Fig. 1).

Heterocyst differentiation involves important morphological changes in which synthesis and remodeling of peptidoglycan play an important role. Two of the validated targets of Yfr1 (*amiC2* and *murC*) are necessary for proper diazotrophic growth (11–13). In addition, *mraY* (also among the targets of Yfr1 validated in this study) has a complex promoter region in which one transcription start site is specifically upregulated in heterocysts (41), suggesting a role of this enzyme in heterocyst differentiation. Strain OE_Yfr1 was unable to grow in liquid medium without combined nitrogen and grew very poorly on solid medium without combined nitrogen (Fig. 5A). Filaments of OE_Yfr1 plated on top of medium without combined nitrogen showed that although the strain was able to differentiate heterocysts with a normal pattern, the heterocyst-vegetative cell connections appeared frequently disrupted (Fig. 5B). These results suggest that the regulation exerted by Yfr1 at the level of a general aspect of the physiology of *Nostoc* sp. PCC 7120 (bacterial envelopes) is also critical for the differentiation of functional mature heterocysts.

**FIG 6:**
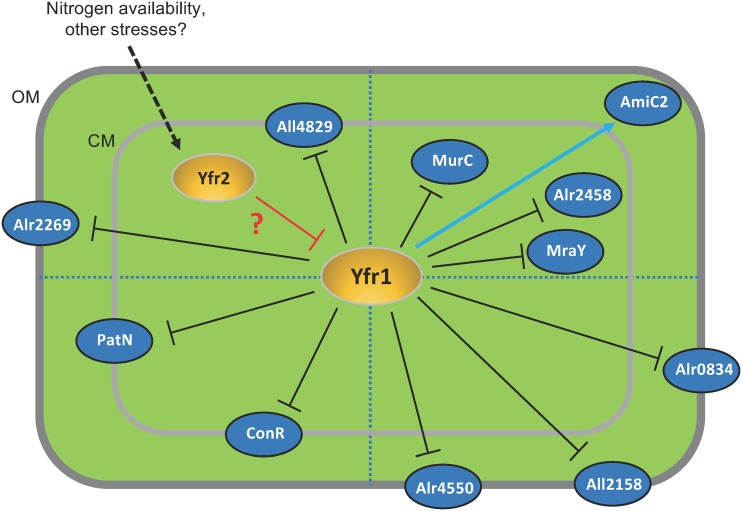
Yfr1 regulatory network. The sRNAs Yfr1 and Yfr2 (orange ovals) and the proteins encoded by Yfr1 target mRNAs (blue ovals) are shown within a schematic drawing of a cell (the thick light-gray or dark-gray lines indicate the cytoplasmic and outer membranes, respectively). Proteins are located in different areas, depending on their functions or the physiological processes in which they may be involved. (Top right) Peptidoglycan biosynthesis or remodeling; (bottom right) transporters; (bottom left) proteins related to heterocyst differentiation; (top left) other functions. Positive regulation is indicated by a blue arrow and negative regulation by black lines with blunt ends. The hypothetical negative regulation of Yfr1 by Yfr2 is indicated by a red line with a blunt end and a red question mark. Possible regulation of Yfr2 by environmental signals is indicated with a dashed arrow. OM, outer membrane; CM, cytoplasmic membrane.

Finally, the expression of most previously studied cyanobacterial sRNAs, such as NsiR1 (42, 43), NsiR4 (44), IsaR1 (45), PsrR1 (46), and NsrR1 (47), transiently changes in response to certain environmental conditions, including light or availability of different nutrients. Expression of Yfr1, however, is relatively high in *Nostoc* sp. PCC 7120 under standard laboratory conditions, and its expression did not significantly change in response to high light or nitrogen availability, the two conditions that we tested (Fig. S1). In Synechococcus elongatus PCC 6301, Yfr1 accumulates up to 18,000 copies per cell, and its expression barely changes under different stresses (26). Yfr1 expression only slightly changes under the tested stress conditions in *Prochlorococcus* MED4 (25). Therefore, the question arises as to how the regulation exerted by Yfr1 on its targets is modulated. One possibility is that the regulatory effects of Yfr1 depend on its controlled sequestration by an RNA binding protein or another sRNA that may act as a trap. In the unicellular cyanobacterium *Prochlorococcus*, the regulation of Yfr1 occurs through sequestration by another conserved sRNA, Yfr2, which contains a conserved region partially complementary to Yfr1 and whose accumulation was found to respond to changes in nitrogen availability (25). In *Nostoc*, Yfr2 is also upregulated under nitrogen deprivation (16), and we have carried out electrophoretic mobility shift assays (EMSAs) showing that *Nostoc* Yfr2 also interacts with Yfr1 in a way similar to that described for *Prochlorococcus* (Fig. S4). Thus, under nitrogen deprivation or other stresses, Yfr2 transcribed from one or several of the four repeats found in the genome of *Nostoc* sp. PCC7120 may bind to the conserved region of Yfr1, preventing the interaction of Yfr1 with its target mRNAs (Fig. 6). Overexpression of Yfr1 in strain OE_Yfr1 might be buffered by its interaction with Yfr2, therefore leading to changes that are less evident than those observed in the E. coli system, in which Yfr2 is absent, while Yfr1 is expressed from a high-copy-number plasmid.

10.1128/mBio.02599-19.4 FIG S4 Interaction between Yfr1 and Yfr2. (A) Clustal Omega alignment of the four Yfr2 RNAs encoded in the genome of *Nostoc* sp. PCC 7120. The region predicted to interact with Yfr1 is shaded in orange. (B) Secondary structures of Yfr1 (black) and Yfr2-1 (red) and their interaction as predicted by RNAcofold. (C) EMSA showing the interaction between *in vitro*-transcribed Yfr1 and Yfr2. Equimolar amounts of Yfr1 and Yfr2 were combined and subjected to electrophoresis as described in Materials and Methods. Download FIG S4, PDF file, 0.2 MB. Copyright © 2020 Brenes-Álvarez et al. 2020 Brenes-Álvarez et al. This content is distributed under the terms of the Creative Commons Attribution 4.0 International license.

The regulatory model proposed here implies that under nitrogen stress, Yfr2 would reduce the inhibitory effect of Yfr1 on a number of proteins required for cell wall changes occurring during heterocyst development. It is possible that additional regulatory mechanisms ensure specific enrichment of those proteins in heterocysts, as discussed above for *mraY*. The results presented here shed light on a general regulatory network that ensures that proper amounts of different proteins related to cell wall biosynthesis and remodeling are present in *Nostoc* cells under different circumstances and specifically during heterocyst development.

The use of regulatory RNAs to control the amounts of outer membrane proteins is well described for enterobacteria (48, 49). Enzymes involved in peptidoglycan biosynthesis have also been found to be controlled by a small RNA (50). Our work shows that also in cyanobacteria, similar mechanisms operate for the coordinated regulation of outer membrane proteins and peptidoglycan biosynthesis enzymes through Yfr1.

Finally, though Yfr1 is universally conserved in cyanobacteria, it appears that different regulatory functions are ascribed to Yfr1 in evolutionarily distant strains. While, in *Prochlorococcus*, Yfr1 regulates genes involved in carbon and nitrogen metabolism (25), here we show that in a heterocyst-forming strain, Yfr1 regulates genes related to cell wall synthesis and remodeling. Because most unicellular cyanobacteria seem to have evolved from nonheterocystous filamentous strains (51), it would be interesting to analyze the targetome of Yfr1 in nonheterocystous filamentous strains.

## MATERIALS AND METHODS

### Strains and growth conditions.

Wild-type *Nostoc* sp. strain PCC 7120 and the OE_C, OE_Yfr1, and OE_as_Yfr1 strains (see Table S2 in the supplemental material) were grown photoautotrophically at 30°C in BG11 medium (52) lacking NaNO_3_ but containing 3.5 mM NH_4_Cl and 7 mM *N*-[Tris(hydroxymethyl)methyl]-2-aminoethanesulfonic acid–NaOH buffer (pH 7.5). For Northern blot analysis of Yfr1 expression under different conditions, cultures of *Nostoc* sp. PCC 7120 were bubbled with an air-CO_2_ mixture (1%, vol/vol) and grown photoautotrophically at 30°C in BG11 medium (52) supplemented with 10 mM NaHCO_3_ (BG11C) lacking NaNO_3_ but containing 6 mM NH_4_Cl and 12 mM *N*-[Tris(hydroxymethyl)methyl]-2-aminoethanesulfonic acid–NaOH buffer (pH 7.5) (BG11C plus NH_4_^+^). To induce nitrogen deficiency, filaments were collected by filtration, washed, and resuspended in nitrogen-free BG11 medium containing 10 mM NaHCO_3_ (BG11_0_C). High-light stress was induced by increasing the light intensity from 50 μE m^−2^ s^−1^ to 500 μE m^−2^ s^−1^.

To test the growth of strains with altered levels of Yfr1 under different conditions, liquid cultures of these strains growing in BG11 media were diluted to an *A*_750_ of 0.17, and 10 μl of serial 5-fold dilutions were spotted on plates containing different nitrogen sources and/or different harmful compounds.

The OE_C, OE_Yfr1, and OE_as_Yfr1 strains were grown in the presence of appropriate antibiotics at the following concentrations: streptomycin (Sm) and spectinomycin (Sp), 2 μg/ml each (liquid medium) or 5 μg/ml each (solid medium).

E. coli strains (Table S2) were grown in LB medium supplemented with appropriate antibiotics (53).

### Generation of *Nostoc* strains with altered levels of Yfr1.

Plasmids and oligonucleotides used in this work are described in Tables S3 and S4, respectively.

10.1128/mBio.02599-19.7 TABLE S3 Plasmids. Download Table S3, DOCX file, 0.02 MB. Copyright © 2020 Brenes-Álvarez et al. 2020 Brenes-Álvarez et al. This content is distributed under the terms of the Creative Commons Attribution 4.0 International license.

10.1128/mBio.02599-19.8 TABLE S4 Oligonucleotides. Download Table S4, DOCX file, 0.02 MB. Copyright © 2020 Brenes-Álvarez et al. 2020 Brenes-Álvarez et al. This content is distributed under the terms of the Creative Commons Attribution 4.0 International license.

We have used pMBA37 (36) as a vector for the overexpression of Yfr1 or Yfr1 in the antisense direction (as_Yfr1). pMBA37 contains the *trc* promoter and the T1 terminator of the *rrnB* gene of E. coli as a transcriptional terminator and allows the overexpression of a cloned sequence flanked by NsiI and XhoI sites. Sequences corresponding to Yfr1 and as_Yfr1 were amplified using genomic DNA as the template and oligonucleotides 575 and 576 or 577 and 578, respectively. After digestion of the PCR products with NsiI and XhoI at the sites provided by the oligonucleotides, the fragments were cloned into NsiI-XhoI-digested pMBA37, rendering pMBA48 (Yfr1) and pMBA49 (as_Yfr1). pMBA51 (a plasmid that overexpresses a control RNA corresponding only to the T1 terminator under the *trc* promoter) (36), pMBA48, and pMBA49 were introduced into *Nostoc* sp. PCC 7120 by conjugation (54), generating strains OE_C (control), OE_Yfr1, and OE_as_Yfr1, respectively (Table S2).

### RNA isolation, Northern blot analysis, and primer extension assays.

Total RNA was isolated using hot phenol as described previously (55), with modifications (27). Northern blot detection of Yfr1 was performed using 10% urea-polyacrylamide gels as described previously (56) and 7.5 μg of total RNA. Northern blot hybridization for mRNAs (*all4316* and *alr5065*) was performed using 1% agarose denaturing formaldehyde gels and 10 μg of total RNA. All RNA samples were then transferred to Hybond-N+ membranes (GE Healthcare) with 20× SSC buffer (1× SSC buffer is 0.15 M NaCl plus 0.015 M sodium citrate). Strand-specific ^32^P-labeled probes were prepared with *Taq* DNA polymerase using a PCR fragment as the template and oligonucleotides specified in Table S4 in a reaction mixture with [α-^32^P]dCTP and a single oligonucleotide as the primer (corresponding to the complementary strand of the sRNA or mRNA to be detected). PCR fragments used as templates for the Yfr1, *all4316*, and *alr5065* probes were amplified from genomic DNA using the oligonucleotide pairs 368/369, 430/431, and 448/449, respectively. Hybridization to *rnpB* (57) or 5S rRNA was used as a loading and transfer control.

### Fluorescent vancomycin conjugate staining.

Filaments from 1 ml of liquid cultures growing in BG11 medium for 5 days were pelleted, washed, resuspended in 50 μl of phosphate-buffered saline (PBS) buffer, mixed thoroughly, and incubated with vancomycin-FL (Van-FL; BODIPY FL conjugate; Invitrogen) or uncoupled vancomycin at 1 μg/ml for 1 h in the dark. After the incubation, unlinked Van-FL or vancomycin was removed by washing the cells twice with PBS buffer. Fluorescence was analyzed using a Leica HCX Plan-APO 63× 1.4-numerical aperture (NA) oil immersion objective attached to a Leica TCS SP2 confocal laser-scanning microscope. Van-FL was excited at 488 nm by an argon ion laser, and the fluorescent emission was monitored in the range of 500 to 530 nm. Samples incubated with uncoupled vancomycin were used to set a threshold to measure the specific fluorescence of Van-FL.

### Computational methods.

Sequences of homologs of Yfr1 were taken from reference 27. CopraRNA (29, 30) was used for the prediction of the targets of Yfr1, with homologs in the genomes of *Nostoc* sp. PCC 7120, *Nostoc* sp. strain PCC 7524, Anabaena variabilis ATCC 29413, *Nostoc* sp. strain PCC 7107, *Calothrix* sp. strain PCC 7507, Nostoc azollae 0708, Nostoc punctiforme PCC 73102, *Calothrix* sp. strain PCC 6303, Cylindrospermum stagnale PCC 7417, and *Anabaena cylindrica* PCC 7122. Prediction of the interaction site between Yfr1 and the 5′-UTRs of several predicted targets in *Nostoc* sp. PCC 7120 was performed using IntaRNA (35). Alignment of Yfr2 homologs was made using Clustal Omega (58). The secondary structures of Yfr1 and Yfr2-1 and their interaction were predicted by RNAcofold (59).

### Reporter assay for *in vivo* verification of targets.

We used the reporter assay described in reference 60 and fusions to the gene encoding superfolder GFP (sfGFP) in plasmid pXG10-SF or pXG30-SF (34) for experimental target verification in E. coli (Table S3). In this system, both the GFP fusions and Yfr1 are transcribed constitutively.

The 5′-UTRs of monocistronic targets were cloned into pXG10-SF from their corresponding transcription start site (according to reference 16) to 42 to 60 nucleotides within the coding region. For targets that could be cotranscribed with a gene located upstream, the last 60 nucleotides of the upstream gene, together with the whole intergenic region plus 30 to 60 nucleotides within the coding region of the target gene, were cloned into pXG30-SF. To facilitate translation in E. coli, GTG start codons were replaced by ATG using overlapping PCR and the oligonucleotides specified in Table S4. PCR fragments containing the region to be cloned were amplified using genomic DNA as the template and oligonucleotides specified in Table S4. Fragments were digested with NsiI and NheI and cloned into pXG10-SF or pXG30-SF treated with the same enzymes, resulting in translational fusions of the different targets to sfGFP (Table S5).

10.1128/mBio.02599-19.9 TABLE S5 Sequences of inserts in the sfGFP plasmids. Download Table S5, DOCX file, 0.02 MB. Copyright © 2020 Brenes-Álvarez et al. 2020 Brenes-Álvarez et al. This content is distributed under the terms of the Creative Commons Attribution 4.0 International license.

To express Yfr1 in E. coli, the sequence encoding Yfr1 was amplified from genomic DNA using primers 422 (5′-end phosphorylated) and 423. The PCR product was digested with XbaI and fused to a plasmid backbone that was amplified from pZE12-luc with primers PLlacOB and PLlacOD (60) and digested with XbaI, rendering pMBA1 (Table S6).

10.1128/mBio.02599-19.10 TABLE S6 Sequences of inserts in plasmids used for expression of Yfr1 and its mutated versions. Download Table S6, DOCX file, 0.02 MB. Copyright © 2020 Brenes-Álvarez et al. 2020 Brenes-Álvarez et al. This content is distributed under the terms of the Creative Commons Attribution 4.0 International license.

For the mutagenesis of Yfr1 and the 5′-UTRs of *all4316* and *alr5065*, mutations were introduced by overlapping PCR with primers containing the desired changes (Table S4), and the fragments were cloned in the same way as the corresponding wild-type versions. The specific mutations were designed based on changes in the hybridization energies predicted by IntaRNA (35).

Combinations of plasmids bearing fragments encoding Yfr1 (or its mutated versions) and the 5′-UTRs of target genes (or mutated versions) were introduced into E. coli DH5α. Plasmid pJV300 was used as a control expressing an unrelated RNA. Fluorescence measurements were done with a microplate reader (Varioskan) using liquid cultures from eight individual colonies of cells carrying each plasmid combination, as previously described (29).

### *In vitro* transcription of RNA and EMSA.

RNAs were transcribed *in vitro* with a MEGAscript high-yield transcription kit (AM1333; Ambion). The DNA templates for the transcription of Yfr1 and Yfr2 were generated by PCR with a primer that includes a T7 promoter sequence upstream of the 5′ end of the corresponding RNA and a primer matching the 3′ end of the RNA (Table S4). The template used for these PCR amplifications was genomic DNA. After *in vitro* transcription, RNAs were treated with DNase I and purified by sequential phenol, phenol-chloroform, and chloroform extractions, ethanol precipitated at −20°C, and washed with 70% ethanol.

For EMSA, 200 ng of each *in vitro*-transcribed Yfr1 and Yfr2 protein were combined in a volume of 5 μl, denatured for 1 min at 95°C, and chilled on ice for 5 min. After denaturing and chilling steps, 10× structure buffer (AM7118; Ambion) was added and the samples were incubated further for 15 min at 37°C before 1 μl of 50% glycerol was added. Samples were run on a 2.5% agarose gel with 0.5% Tris-borate-EDTA (TBE) buffer at 50 V in a cold chamber.
