# Supplementary material for: The Integrity of the Cell Wall and Its Remodeling during Heterocyst Differentiation Are Regulated by Phylogenetically Conserved Small RNA Yfr1 in Nostoc sp. Strain PCC 7120
Source: mBio. 2020 Jan 21;11(1):e02599-19. doi: 10.1128/mBio.02599-19 (PMC6974561; doi:10.1128/mBio.02599-19)
Supplement: FIG S1 [file mBio.02599-19-sf001.pdf]

Figure S1

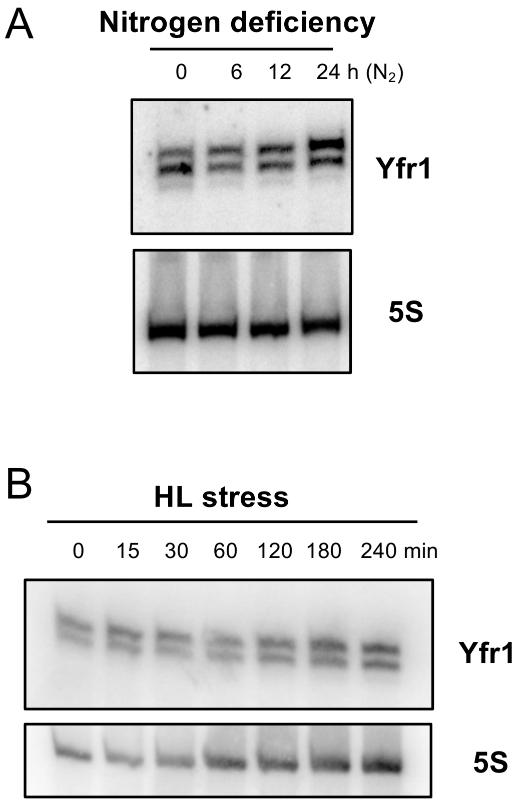

Figure S1. Expression of Yfr1 in *Nostoc* sp. PCC 7120. (A) Northern blot analysis with total RNA extracted at different times after removal of combined nitrogen. (B) Northern blot analysis with total RNA from cells growing in the presence of ammonia at 50 µE m^{-2} s^{-1} and incubated at 500 µE m^{-2} s^{-1} (high light stress, HL) for the indicated times. All membranes were hybridized with probes for Yfr1 and 5S RNA as loading control.
