# Supplementary material for: The Integrity of the Cell Wall and Its Remodeling during Heterocyst Differentiation Are Regulated by Phylogenetically Conserved Small RNA Yfr1 in Nostoc sp. Strain PCC 7120
Source: mBio. 2020 Jan 21;11(1):e02599-19. doi: 10.1128/mBio.02599-19 (PMC6974561; doi:10.1128/mBio.02599-19)
Supplement: TABLE S1 [file mBio.02599-19-st001.docx]

**Table S1.** Top 50 predicted targets of Yfr1.

| **Gene** | **Annotation^a^** | **fdr** | **p-value** |
| --- | --- | --- | --- |
| *alr2458* | alanine racemase | 7,52E-09 | 2,14E-12 |
| *alr4550* | porin-like, OprB-like | 1,51E-08 | 8,60E-12 |
| *alr5065* (*murC*) | UDP-N-acetylmuramate-L-alanine ligase | 2,82E-08 | 2,40E-11 |
| *all4829* | undecaprenyl-phosphate galactosephosphotransferase | 2,64E-07 | 3,00E-10 |
| *all2158* | ferrichrome-iron receptor (TonB-dependent transporter) | 3,29E-07 | 4,68E-10 |
| *all4316* (*mraY*) | phospho-N-acetylmuramoyl-pentapeptide transferase | 5,12E-07 | 8,72E-10 |
| *alr2269* | chloroplastic outer envelope membrane protein homolog, Omp85, Toc75 | 6,58E-07 | 1,31E-09 |
| *all5094* | oxidoreductase | 7,30E-07 | 1,66E-09 |
| *all1835* | unknown protein | 2,39E-06 | 6,11E-09 |
| *alr0834* | porin, ortholog of PMM1119 and PMM1121 | 3,05E-06 | 8,65E-09 |
| *all4499* | porin-like, OprB-like | 4,93E-06 | 1,54E-08 |
| *all2352* | WD-40 repeat protein. | 6,88E-06 | 2,35E-08 |
| *alr4077* | hypothetical protein | 7,09E-06 | 2,62E-08 |
| *all0187* (*conR*) | LytR-CpsA-Psr superfamily | 9,05E-06 | 3,68E-08 |
| *all1587* | hypothetical protein | 9,05E-06 | 3,86E-08 |
| *all0481* | unknown protein | 9,32E-06 | 4,24E-08 |
| *alr0068* | adenylate kinase | 1,37E-05 | 6,85E-08 |
| *all4220* | hypothetical protein | 1,37E-05 | 6,99E-08 |
| *alr3380* | dolichol-phosphate mannosyltransferase | 3,32E-05 | 1,81E-07 |
| *alr3654* | uncharacterized RNA methyltransferase | 3,32E-05 | 1,89E-07 |
| *alr2887* (*hgdD*) | outer membrane channel, TolC-like, | 4,54E-05 | 2,78E-07 |
| *alr2057* (*aroE*) | shikimate 5-dehydrogenase | 4,54E-05 | 2,96E-07 |
| *alr1805* | hypothetical protein | 4,54E-05 | 2,97E-07 |
| *all3826* | peptidoglycan-binding protein | 4,92E-05 | 3,36E-07 |
| *alr4812* (*patN*) | heterocyst differentiation related protein | 5,49E-05 | 3,90E-07 |
| *all4026* | similar to TonB-dependent receptor | 7,32E-05 | 5,41E-07 |
| *asr3279* | unknown protein | 8,67E-05 | 6,65E-07 |
| *alr0093* (*amiC2, hcwA*) | N-acetylmuramoyl-L-alanine amidase | 9,51E-05 | 7,57E-07 |
| *alr5049* | unknown protein, putative OmpA/MotB system | 9,87E-05 | 8,13E-07 |
| *all3669* | unknown protein | 1,05E-04 | 8,97E-07 |
| *alr4123* (*prk*) | phosphoribulokinase | 1,40E-04 | 1,23E-06 |
| *all1340* | unknown protein | 1,44E-04 | 1,31E-06 |
| *alr5030* | hypothetical protein | 1,99E-04 | 1,86E-06 |
| *all3902* | hypothetical protein | 2,35E-04 | 2,27E-06 |
| *all2116* | hypothetical protein | 3,54E-04 | 3,58E-06 |
| *alr4602* | type I site-specific deoxyribonuclease chain S | 3,54E-04 | 3,62E-06 |
| *alr2479* | LytR-CpsA-Psr superfamily | 3,80E-04 | 4,00E-06 |
| *alr4714* | unknown protein, specific to heterocyst clade | 3,85E-04 | 4,15E-06 |
| *alr4269* | hypothetical protein | 3,91E-04 | 4,33E-06 |
| *alr3004* | unknown protein | 4,43E-04 | 5,03E-06 |
| *all0089* | hypothetical protein | 4,59E-04 | 5,34E-06 |
| *all0571* | cyanophycinase | 4,77E-04 | 5,69E-06 |
| *all0211* | hypothetical protein | 5,09E-04 | 6,21E-06 |
| *asl4743* | unknown protein, specific to heterocyst clade | 5,36E-04 | 6,70E-06 |
| *alr3955* | thioredoxin | 6,42E-04 | 8,21E-06 |
| *all3310* | hypothetical outer membrane receptor for ferritin, (TonB-dependent transporter) | 6,66E-04 | 8,71E-06 |
| *all0997* | unknown protein, specific to heterocyst clade | 7,50E-04 | 1,00E-05 |
| *all4106* | probable oxidoreductase | 7,91E-04 | 1,08E-05 |
| *alr0255* | unknown protein, specific to heterocyst clade | 7,98E-04 | 1,11E-05 |

^a^Annotation based on NCBI (https://www.ncbi.nlm.nih.gov/), Cyanobase (http://genome.microbedb.jp/cyanobase/), CopraRNA results (1, 2) and previous studies.

Interactions verified in this work are highlighted in gray.

**References:**

1. **Wright PR, Georg J, Mann M, Sorescu DA, Richter AS, Lott S, Kleinkauf R, Hess WR, Backofen R.** 2014. CopraRNA and IntaRNA: predicting small RNA targets, networks and interaction domains. Nucleic Acids Res **42:**W119-W123.

2. **Wright PR, Richter AS, Papenfort K, Mann M, Vogel J, Hess WR, Backofen R, Georg J.** 2013. Comparative genomics boosts target prediction for bacterial small RNAs. Proc Natl Acad Sci U S A **110:**E3487-3496.
