# Supplementary material for: The Integrity of the Cell Wall and Its Remodeling during Heterocyst Differentiation Are Regulated by Phylogenetically Conserved Small RNA Yfr1 in Nostoc sp. Strain PCC 7120
Source: mBio. 2020 Jan 21;11(1):e02599-19. doi: 10.1128/mBio.02599-19 (PMC6974561; doi:10.1128/mBio.02599-19)
Supplement: FIG S2 [file mBio.02599-19-sf002.pdf]

Figure S2

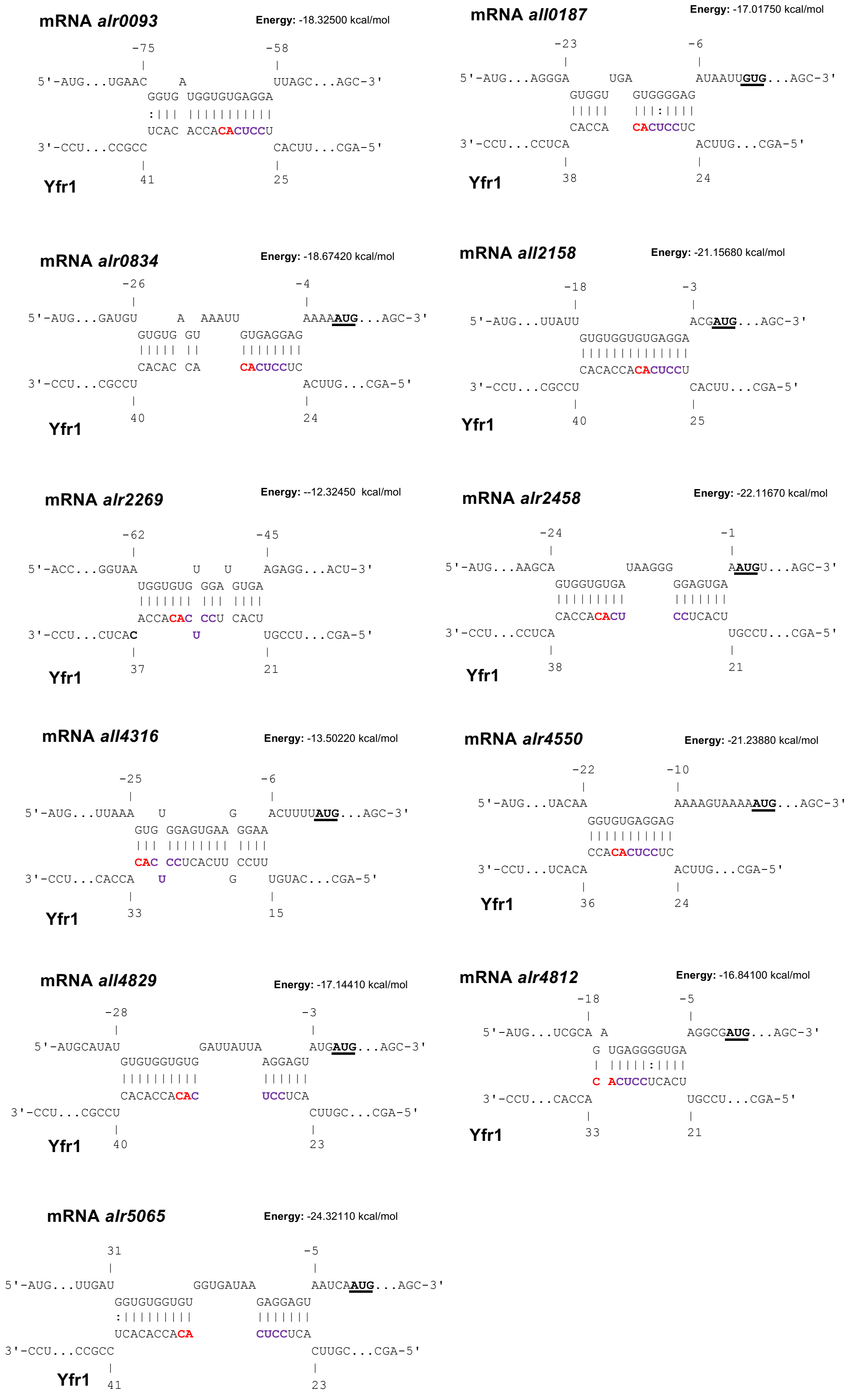

Figure S2. Interactions predicted by IntaRNA between Yfr1 and several mRNAs. Nucleotides of 5'UTRs are numbered with respect to the start of the coding sequence (start codons are indicated in bold and underlined). Positions of the mutations introduced in Yfr1_UG are shown in red. Positions of the mutations introduced in Yfr1_AAAA are shown in purple.
