# Supplementary material for: The Integrity of the Cell Wall and Its Remodeling during Heterocyst Differentiation Are Regulated by Phylogenetically Conserved Small RNA Yfr1 in Nostoc sp. Strain PCC 7120
Source: mBio. 2020 Jan 21;11(1):e02599-19. doi: 10.1128/mBio.02599-19 (PMC6974561; doi:10.1128/mBio.02599-19)
Supplement: TABLE S2 [file mBio.02599-19-st002.docx]

**Table S2.** Strains

| **Strain** | **Description** | **Reference** |
| --- | --- | --- |
| ***Escherichia coli*** |  |  |
| DH5α | Used for routine transformation | (1) |
|  |  |  |
| ***Nostoc* sp.** |  |  |
| PCC 7120 | Wild type | Pasteur Culture Collection |
| OE_C | Sm^R^Sp^R^, pMBA51 inserted in plasmid alpha. T1 terminator of *E.coli* *rrnB* gene expressed constitutively from *trc*  promoter. | (2) |
| OE_Yfr1 | Sm^R^Sp^R^, pMBA48 inserted in plasmid alpha. Yfr1 expressed constitutively from *trc*  promoter. | This work |
| OE_as_Yfr1 | Sm^R^Sp^R^, pMBA49 inserted in plasmid alpha.  Antisense to Yfr1 expressed constitutively from *trc*  promoter. | This work |

**References:**

1. **Hanahan D.** 1983. Studies on transformation of *Escherichia coli* with plasmids. J Mol Biol **166:**557-580.

2. **Olmedo-Verd E, Brenes-Álvarez M, Vioque A, Muro-Pastor AM.** 2019. A heterocyst-specific antisense RNA contributes to metabolic reprogramming in *Nostoc* sp. PCC 7120. Plant Cell Physiol **60:**1646-1655.
