# Supplementary material for: The Integrity of the Cell Wall and Its Remodeling during Heterocyst Differentiation Are Regulated by Phylogenetically Conserved Small RNA Yfr1 in Nostoc sp. Strain PCC 7120
Source: mBio. 2020 Jan 21;11(1):e02599-19. doi: 10.1128/mBio.02599-19 (PMC6974561; doi:10.1128/mBio.02599-19)
Supplement: FIG S3 [file mBio.02599-19-sf003.pdf]

Figure S3

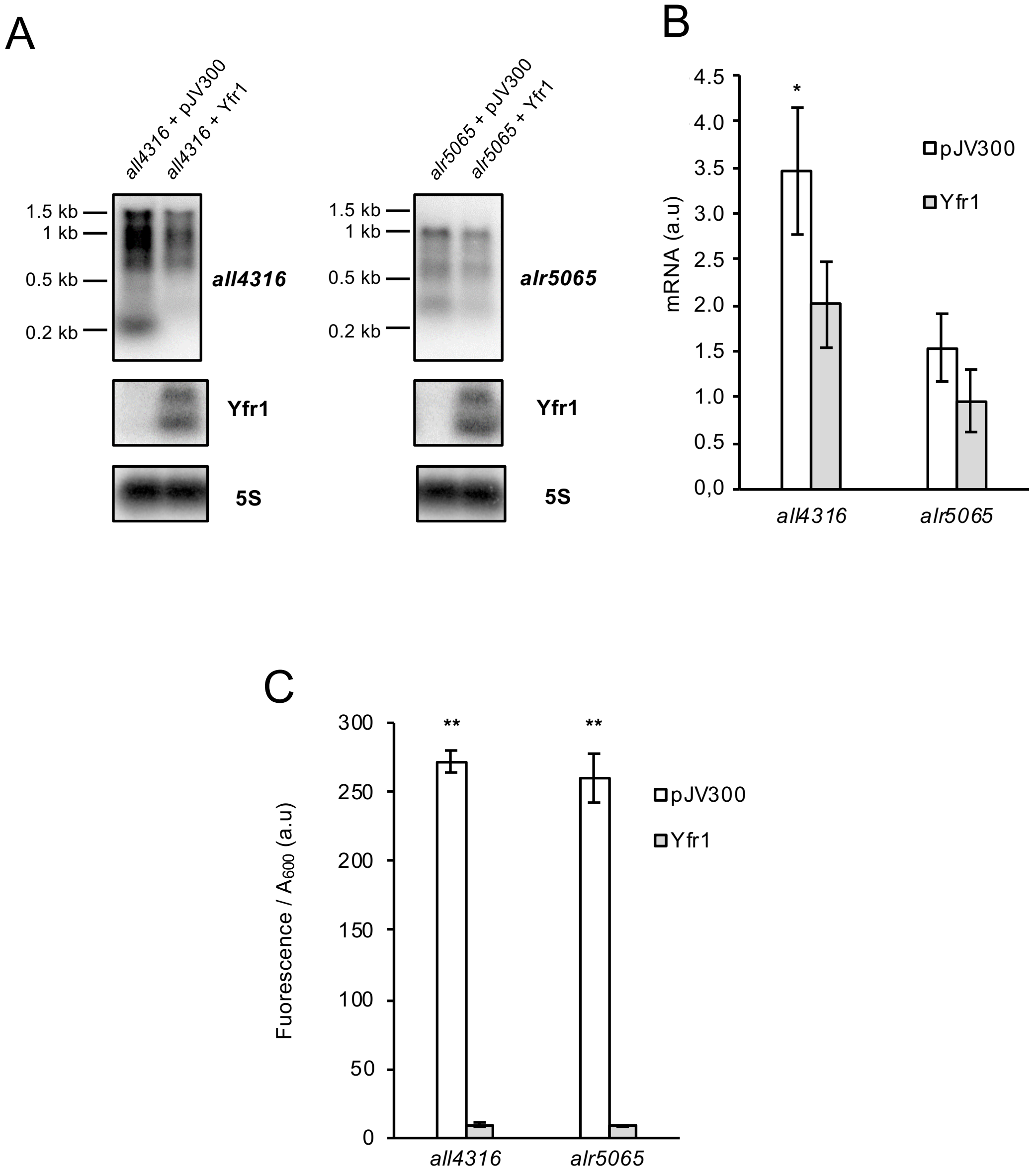

Figure S3. Comparison of the effects of Yfr1 on expression of *all4316* and *alr5065* at the mRNA (A, B) and protein level (C) in *E. coli*. (A) Northern blot with RNA isolated from *E. coli* cells bearing plasmid pMBA4 (*all4316-sfgfp*) or pMBA7 (*alr5065-sfgfp*) and a plasmid expressing Yfr1 (pMBA1) or a control RNA (pJV300) hybridized with probes for *all4316*, *alr5065*, Yfr1 and 5S RNA as loading control. (B) Quantification of signals in Northern blots. Data are presented as the mean +/- standard deviation of the signal normalized to the 5S signal (three individual experiments). (C) Fluorescence of the *E. coli* cultures analyzed in (A-B).The data are presented as mean +/- standard deviation of the results from three cultures after subtraction of fluorescence in cells bearing pXG0 and normalized for cell density (A_{600}). (*) p<0,05: (**) p<0,0001, t-test.
