# Supplementary material for: The Integrity of the Cell Wall and Its Remodeling during Heterocyst Differentiation Are Regulated by Phylogenetically Conserved Small RNA Yfr1 in Nostoc sp. Strain PCC 7120
Source: mBio. 2020 Jan 21;11(1):e02599-19. doi: 10.1128/mBio.02599-19 (PMC6974561; doi:10.1128/mBio.02599-19)
Supplement: FIG S4 [file mBio.02599-19-sf004.pdf]

Figure S4

A

```
Yfr2_1 --GUGUGAGGAGCAAGUUGAAACUAAAAAGCAUCUGGGGUGGAAACACGGCAACACUCCCAGAUGCUUUUU----- 69
Yfr2_2 GUGUGUGAGGAGCAAGUUAAAAAUAAAAAGCGUCUGGGGUGGAAACACGGCAACACUCCCAGGCGCUUUUU----- 71
Yfr2_3 GUGUGUGAGGAGCAAGUUAAAAAUAAAAAGCGUCUGGGGUGGAAACACGGCAACACUCCCAGGCGCUUUUUU---- 72
Yfr2_4 --GUGUGAGGAGCAAGUUGAAAUUA-AGAGCGCUUAGAGUGGAAACACGGUAACACUUCUGAGCGCUUUUUAAUUU 73
         **************** *** ** * ***   * * ************ ****** *      *******
```

B

C

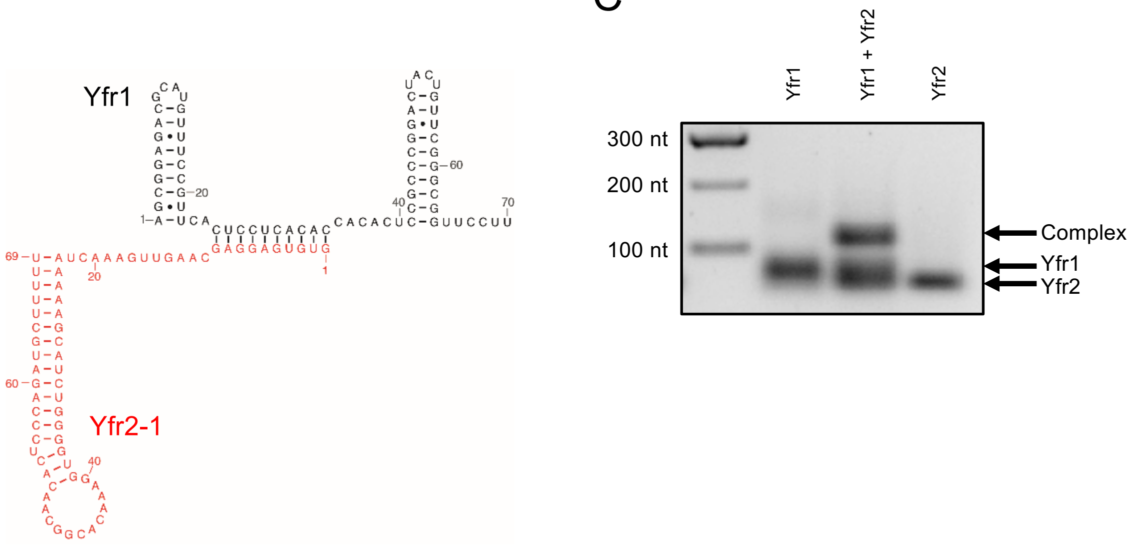

Figure S4. Interaction between Yfr1 and Yfr2. (A) Clustal Omega alignment of the four Yfr2 RNAs encoded in the genome of *Nostoc* sp. PCC 7120. The region predicted to interact with Yfr1 is shaded in orange. (B) Secondary structures of Yfr1 (black) and Yfr2-1 (red) and their interaction as predicted by RNAcofold. (C) EMSA showing the interaction between *in vitro* transcribed Yfr1 and Yfr2. Equimolar amounts of Yfr1 and Yfr2 were combined and subjected to electrophoresis as described in the Material and Methods.
