# Supplementary material for: The Integrity of the Cell Wall and Its Remodeling during Heterocyst Differentiation Are Regulated by Phylogenetically Conserved Small RNA Yfr1 in Nostoc sp. Strain PCC 7120
Source: mBio. 2020 Jan 21;11(1):e02599-19. doi: 10.1128/mBio.02599-19 (PMC6974561; doi:10.1128/mBio.02599-19)
Supplement: TABLE S3 [file mBio.02599-19-st003.docx]

**Table S3.** Plasmids

| **Name** | **Description** | **Reference** |
| --- | --- | --- |
| pMBA37 | Ap^R^Sm^R^Sp^R^, plasmid for the overexpression of transcripts from the *trc* promoter and followed by the *T1* terminator of *E.coli rrnB* gene, used as transcriptional terminator. | (1) |
| pMBA51 | Ap^R^Sm^R^Sp^R^, Control plasmid, expresses a 56 nt transcript derived from the *T1* terminator of *E.coli* *rrnB* gene from *trc* promoter. | (1) |
| pMBA48 | Ap^R^Sm^R^Sp^R^, plasmid based on pMBA37 for the overexpression of Yfr1. | This work |
| pMBA49 | Ap^R^Sm^R^Sp^R^, plasmid based on pMBA37 for the overexpression of an antisense to Yfr1. | This work |
| pJV300 | Control plasmid, expresses a ~50 nt transcript  derived from *T1* terminator of *E.coli rrnB* gene. | (2) |
| pXG0 | Cm^R^, control plasmid without *sfgfp.* | (3) |
| pXG10-SF | Cm^R^, plasmid for construction of translational  sfGFP fusions of monocistronic targets. | (4) |
| pXG30-SF | Cm^R^, plasmid for construction of translational  sfGFP fusions of dicistronic targets. | (4) |
| pZE12-luc | Ap^R^, plasmid to express sRNAs under control of P_Llac-O_ promoter. | (5) |
| pMBA1 | Ap^R^, plasmid based on pZE12-luc expressing Yfr1. | This work |
| pMBA13 | Ap^R^, same as pMBA1 but with AC to UG change at positions 31-32 of Yfr1 (mut 31_32). | This work |
| pMBA15 | Ap^R^, same as pMBA1 but with CCUC to AAAA change at positions 27-30 of Yfr1 (mut AAAA). | This work |
| pMBA2 | Cm^R^, sfGFP reporter plasmid based on pXG10-SF containing the *all0187* 5’UTR plus sequences encoding the first 20 aminoacids (with mutation in start codon GTG -> ATG). | This work |
| pMBA3 | Cm^R^, sfGFP reporter plasmid based on pXG30-SF containing the *all2158* 5’UTR plus sequences encoding the first 20 aminoacids. | This work |
| pMBA4 | Cm^R^, sfGFP reporter plasmid based on pXG30-SF containing the *all4316* 5’UTR plus sequences encoding the first 20 aminoacids (with mutation in start codon GTG -> ATG). | This work |
| pMBA5 | Cm^R^, sfGFP reporter plasmid based on pXG10-SF containing the *all4829* 5’UTR plus sequences encoding the first 17 aminoacids. | This work |
| pMBA6 | Cm^R^, sfGFP reporter plasmid based on pXG30-SF containing the *alr0093* 5’UTR plus sequences encoding the first 20 aminoacids (with mutation in start codon GTG -> ATG). | This work |
| pMBA7 | Cm^R^, sfGFP reporter plasmid based on pXG10-SF containing the *alr5065* 5’UTR plus sequences encoding the first 20 aminoacids. | This work |
| pMBA8 | Cm^R^, sfGFP reporter plasmid based on pXG10-SF containing the *alr2458* 5’UTR plus sequences encoding the first 14 aminoacids. | This work |
| pMBA9 | Cm^R^, sfGFP reporter plasmid based on pXG10-SF containing the *alr4550* 5’UTR plus sequences encoding the first 20 aminoacids. | This work |
| pMBA10 | Cm^R^, sfGFP reporter plasmid based on pXG10-SF containing the *alr4812* 5’UTR plus sequences encoding the first 20 aminoacids. | This work |
| pMBA11 | Cm^R^, sfGFP reporter plasmid based on pXG10-SF containing the *alr0834* 5’UTR plus sequences encoding the first 20 aminoacids. | This work |
| pMBA17 | Cm^R^, same as pMBA7 but with a GT to CA change  at position -21 and -22 of the 5'-UTR with respect to the start codon (*alr5065* mut). | This work |
| pMBA19 | Cm^R^, same as pMBA4 but with a GT to CA change  at position -22 and -23 of the 5'-UTR with respect to the start codon (*all4316* mut). | This work |
| pMBA90 | Cm^R^, sfGFP reporter plasmid based on pXG10-SF containing the *alr2269* 5’UTR plus sequences encoding the first 20 aminoacids (with mutation in start codon GTG -> ATG). | This work |

**References:**

1. **Olmedo-Verd E, Brenes-Álvarez M, Vioque A, Muro-Pastor AM.** 2019. A heterocyst-specific antisense RNA contributes to metabolic reprogramming in *Nostoc* sp. PCC 7120. Plant Cell Physiol **60:**1646-1655.

2. **Sittka A, Pfeiffer V, Tedin K, Vogel J.** 2007. The RNA chaperone Hfq is essential for the virulence of *Salmonella typhimurium*. Mol Microbiol **63:**193-217.

3. **Urban JH, Vogel J.** 2009. A green fluorescent protein (GFP)-based plasmid system to study post-transcriptional control of gene expression in vivo. Methods Mol Biol **540:**301-319.

4. **Corcoran CP, Podkaminski D, Papenfort K, Urban JH, Hinton JC, Vogel J.** 2012. Superfolder GFP reporters validate diverse new mRNA targets of the classic porin regulator, MicF RNA. Mol Microbiol **84:**428-445.

5. **Lutz R, Bujard H.** 1997. Independent and tight regulation of transcriptional units in *Escherichia coli* via the LacR/O, the TetR/O and AraC/I1-I2 regulatory elements. Nucleic Acids Res **25:**1203-1210.
