# Supplementary material for: The Integrity of the Cell Wall and Its Remodeling during Heterocyst Differentiation Are Regulated by Phylogenetically Conserved Small RNA Yfr1 in Nostoc sp. Strain PCC 7120
Source: mBio. 2020 Jan 21;11(1):e02599-19. doi: 10.1128/mBio.02599-19 (PMC6974561; doi:10.1128/mBio.02599-19)
Supplement: TABLE S4 [file mBio.02599-19-st004.docx]

**Table S4.** Oligonucleotides

| **Name** | **Sequence (5’-3’)** | **Used for** |
| --- | --- | --- |
| 189 | CGCACTGACCGAATTCATTAA | Amplification of plasmid backbone from pZE12-luc |
| 190 | GTGCTCAGTATCTTGTTATCCG |  |
| 356 | AATCAGAACGCAGAAGCGGT | Probe for 5S of *E. coli* |
| 357 | CGGCGGATTTGTCCTACTCA |  |
| 368 | aaagggaatagggaacggcg | Probe for Yfr1 |
| 369 | cggctgctgacatgcctaaa |  |
| 422 | 5’P-AGCGGAGACGCATGTTTCCGTTCAC | Cloning of Yfr1 in pZE12-luc |
| 423 | GTTTTTTCTAGATACATTTGCAAAGAAGCTAAAGAATT |  |
| 424 | GTTTTATGCATAAACGGTAGAACATTTCAAGTG | Cloning of *all0187* 5’-UTR with mutation in start codon (GTG->ATG) in pXG10-SF |
| 425 | GTTTTGCTAGCGAGATCCGGTATTTGTTGAGGC |  |
| 426 | TTAATTACAATTATCTCCCCACTCAACCA |  |
| 427 | TAATTATGATTAAACAGGTTCAATGGTCA |  |
| 428 | GTTTTATGCATGCAGGTCACAAGACGGTTTT | Cloning of *all2158* 5’-UTR in pXG30-SF |
| 429 | GTTTTGCTAGCTCCACAAAGGCTGATGATAA |  |
| 430 | GTTTTATGCATGACGACGATATCACCATAGA | Cloning of all*4316* 5’-UTR with mutation in start codon (GTG->ATG) in pXG30-SF |
| 431 | GTTTTGCTAGCAGAAGCTAGACCAATACCAG |  |
| 432 | GCGTCCATAAAAGTTTCCCTTCACTCCAC |  |
| 433 | CTTTTATGGACGCTAAATTATCGCCTA |  |
| 434 | GTTTTATGCATATGTGTGGTGTGGATTATTAAGG | Cloning of *all4829* 5’-UTR in pXG10-SF |
| 435 | GTTTTGCTAGCATCTTGCCGTAAGCGTCGTTT |  |
| 436 | GTTTTATGCATCTTCAGTACCTCAAACGATAG | Cloning of *alr0093* 5’-UTR with mutation in start codon (GTG->ATG) in pXG30-SF |
| 437 | GTTTTGCTAGCTGGTGACGATAGTAAGAAGA |  |
| 438 | AATTTCATAATCTTTTCTCCTGATGTGCG |  |
| 439 | AGATTATGAAATTACACTGGTTACTATC |  |
| 440 | GTTTTATGCATATACTTTTGAAGTAAATATTACTTC | Cloning of *all4829* 5’-UTR in pXG10-SF |
| 441 | GTTTTGCTAGCATTTGTTTCTGTTATGGCAG |  |
| 442 | GTTTTATGCATGTAATTGATCCTGTCGGTTA | Cloning of *alr2458*  5’-UTR in pXG10-SF |
| 443 | GTTTTGCTAGCTTGATGAGAAGCCATACTTG |  |
| 444 | GTTTTATGCATATATGGGTACGTTTTTGCAT | Cloning of *alr4550* 5’-UTR in pXG10-SF |
| 445 | GTTTTGCTAGCTAGTGTTGCGCCTAAAAC |  |
| 446 | GTTTTATGCATATCAGAATATTCATCTGTAAAAAA | Cloning of *alr4812* 5’-UTR in pXG10-SF |
| 447 | GTTTTGCTAGCCAAGAGAGCCGCCAGTCCAG |  |
| 448 | GTTTTATGCATGTTATTTGTGATTGATGGTGTG | Cloning of *alr5065* 5’-UTR in pXG10-SF |
| 449 | GTTTTGCTAGCTATACCGCCGATTCCGATAA |  |
| 470 | CTCCTCTGACCACACTCCGCCCGGACTA | Mutagenesis of Yfr1 to generate Yfr1_UG |
| 471 | TGTGGTCAGAGGAGTGAACGGAAACATG |  |
| 484 | ATTAAACAGTGGAGTGAAGGGAAACTTT | Mutagenesis of *all4316* 5’-UTR to generate *all4316* mut |
| 485 | CTCCACTGTTTAATAAAACTGAATTATA |  |
| 486 | TGTGGTCAGGTGATAAGAGGAGTAATCA | Mutagenesis of *alr5065* 5’-UTR to generate *alr5065* mut |
| 487 | ATCACCTGACCACACCATCAATCACAAA |  |
| 491 | TCACTAAAAACACCACACTCCGCCCGGAC | Mutagenesis of Yfr1 to generate Yfr1_AAAA |
| 492 | GGTGTTTTTAGTGAACGGAAACATGCGTC |  |
| 575 | GTTTTATGCATAGCGGAGACGCATGTTTCC | Cloning of Yfr1 in pMBA37 |
| 576 | GTTTTCTCGAGGGAACCGCCCGAACAGTAGT |  |
| 577 | GTTTTATGCATGGAACCGCCCGAACAGTAGT | Cloning of as_Yfr1 in pMBA37 |
| 578 | GTTTTCTCGAGAGCGGAGACGCATGTTTCCG |  |
| 874 | GTTTTATGCATacctttggcataaacaagcaat | Cloning of *alr2269* 5’-UTR with mutation in start codon (GTG->ATG) in pXG10-SF |
| 875 | GTTTTGCTAGCagtttgggcatttgcagttaatga |  |
| 876 | cagccgccaTtaatacgggagataaacgca |  |
| 877 | tcccgtattaAtggcggctgtagcaatcac |  |
| 903 | TAATACGACTCACTATAGGGAGCGGAGACGCATGTTTCCG | PCR of template for *in vitro* transcription of Yfr1 |
| 904 | AAGGAACCGCCCGAACAGTAG |  |
| 905 | TAATACGACTCACTATAGGGTGTGAGGAGCAAGTTGAAA | PCR of template for *in vitro* transcription of Yfr2 |
| 906 | AAAAAGCATCTGGGAGTGTTGC |  |

Restriction sites used for cloning (underlined), point mutations introduced (red) and 5’ monophosphate (5’P) are indicated.
