# Supplementary material for: The Integrity of the Cell Wall and Its Remodeling during Heterocyst Differentiation Are Regulated by Phylogenetically Conserved Small RNA Yfr1 in Nostoc sp. Strain PCC 7120
Source: mBio. 2020 Jan 21;11(1):e02599-19. doi: 10.1128/mBio.02599-19 (PMC6974561; doi:10.1128/mBio.02599-19)
Supplement: TABLE S5 [file mBio.02599-19-st005.docx]

**Table S5.** Sequences of inserts in the sfGFP fusion plasmids.

| **Plasmid** | **Sequence** | **Description** |
| --- | --- | --- |
| pMBA2 | atgcatAAACGGTAGAACATTTCAAGTGTACAGTGCGGTCTTACACTGGCAAGATTGGTAGCTTGCTTTAACATAAATTTTAGGGAGTGGTTGAGTGGGGAGATAATTATGATTAAACAGGTTCAATGGTCAGATAATCAGGTCGCGCCTCAACAAATACCGGATCTCgctagc | *all0187* (mutation in start codon) |
| pMBA3 | atgcatGCAGGTCACAAGACGGTTTTTGGATCATAACTGGCGGTTTTTGGATAGACACCCTGCGAGTAACTTCCCTATACTACTTTAACTGCTATTAAGAATTATTAGCAGTAATAGGCTGACTTTGATAAGTTTATTGTGTGGTGTGAGGAACGATGAAGCGTTGGTGTTTGTCCCCCAGTATTCATTTATGGTTTATCATCAGCCTTTGTGGAgctagc | *all2158* |
| pMBA4 | atgcatGACGACGATATCACCATAGACGAAGATTAGTCTTTTGAGACTGGGACTCTGTTATATATAGTCCCTATAATTCAGTTTTATTAAAGTGTGGAGTGAAGGGAAACTTTTATGGACGCTAAATTATCGCCTAATCAAGGATTAAATATTTCTGGTATTGGTCTAGCTTCTgctagc | *all4316*  (mutation in start codon) |
| pMBA5 | atgcatATGTGTGGTGTGGATTATTAAGGAGTATGATGACTGCCCAGAGTTCACTCCTCTCCGGCAAACGACGCTTACGGCAAGATgctagc | *all4829* |
| pMBA6 | atgcatCTTCAGTACCTCAAACGATAGATAATCAGTCAGTCTTCACAAACTTATAATCATCCTGATTGCCACACTATCCCTGTTATTCCTGAAGATTTTGTGTGAATACTAACCACATCCTTATATTCTGAACCTGGATCTTATGCCTTCACGACATGGCAAAATCAAAAGGGCGTGTTAAGATGTGTCGCCATTAGTTGTATTTTTATACCAGCGCAAAATTTTTATTGCAGTAAAGCTGAAGTTTGAACGGTGATGGTGTGAGGATTAGCCAACATTTTGGAGCCTTAACGGCTCTCATGAACGCACATCAGGAGAAAAGATTATGAAATTACACTGGTTACTATCGTCACCAgctagc | *alr0093*  (mutation in start codon) |
| pMBA7 | atgcatGTTATTTGTGATTGATGGTGTGGTGTGGTGATAAGAGGAGTAATCAATGAATAATAGTGTAGATTTTGGCGGTAGACCATTTCATTTTATCGGAATCGGCGGTATAgctagc | *alr5065* |
| pMBA8 | atgcatGTAATTGATCCTGTCGGTTATTGGTAATGGCTCAGAACCCCTATTAATTGGCTATTACCTCAGTCAAAAACAGAAGTTTAGCTCTTATTACTGCTAGTTATTAGTAGCAAAAAGAACCTGTTAATTTTAATTAAGAGGTGGAGTATTGAGTAAAATACACTAGGTTCCAGTCTCTAACCCCCTCCCTGATCAACATCTGAACGAGAAAATTTCAAAATTTTACTCAAGTATGTTGTATCTGTTGCGAAATGCGAGTTAAACTTCCTGATTAACTCTGATGTGGCTATAAAACCAATTCGCAATAGCCTGCGGCAAGGCTAACGCCAACGCAATTCGGAATAAATACGCATTTAAAAAGCAGTGGTGTGATAAGGGGGAGTGAAATGTTAAGTCGCCAACAAGCCTCAAGTATGGCTTCTCATCAAgctagc | *alr2458* |
| pMBA9 | atgcatATATGGGTACGTTTTTGCATAAGTTAATTGCATTAATTTTTAATACTAAGTTAATCAATCATCAGCTTGGCAAAAACTAAAATTGTAATAGTGGCAACTATGCCTCATCAAAAGATTTCAATAAGATGTCACATTTTTGAGAGTAGCTGCAACAAGAACAATTATCTATACAAGGTGTGAGGAGAAAAGTAAAAATGTCTAATCTATTGTGGAAATCCCTAGTGGTTAGCCCTGCTGTTTTAGGCGCAACACTAgctagc | *alr4550* |
| pMBA10 | atgcatATCAGAATATTCATCTGTAAAAAAACTAGCTGAAACAGCAGCTTAATATTTTGGGTATTATGTGAATTAAGTAGGCTAAAAGATTATGAGTGTGAGAGTAGGTCAGTCAAACGTTATGGAATTTACATCGCAGATGAGGGGTGAAGGCGATGAGTGAAAGTATGGCATTTATCGGCGGTGTCGCCGTAGCTGGACTGGCGGCTCTCTTGgctagc | *alr4812* |
| pMBA11 | atgcatATACTTTTGAAGTAAATATTACTTCAAATAAACTTGGTAAATCTATTGCCAAGAAATAAGATGTGTGTGAGTAAATTGTGAGGAGAAAAATGCAGAAAATATGGAAGTATTGGCTGGTTAACCCAGTAATTTGCAGCACAATGCTATTTTCTGGCGCTGCTGCCTTTGCAGGAGAAACCCCTGCCATAACAGAAACAAATgctagc | *alr0834* |
| pMBA17 | atgcatGTTATTTGTGATTGATGGTGTGGTCAGGTGATAAGAGGAGTAATCAATGAATAATAGTGTAGATTTTGGCGGTAGACCATTTCATTTTATCGGAATCGGCGGTATAgctagc | *alr5065* mut |
| pMBA19 | atgcatGACGACGATATCACCATAGACGAAGATTAGTCTTTTGAGACTGGGACTCTGTTATATATAGTCCCTATAATTCAGTTTTATTAAACAGTGGAGTGAAGGGAAACTTTTATGGACGCTAAATTATCGCCTAATCAAGGATTAAATATTTCTGGTATTGGTCTAGCTTCTgctagc | *all4316* mut |
| pMBA90 | atgcatACCTTTGGCATAAACAAGCAATTGCCTGGTAATGGTGTGTGGATGTGAAGAGGAAGAAAGGAATTAAAGACGATGCGTTTATCTCCCGTATTAATGGCGGCTGTAGCAATCACAGCACCCTTGAGTAGTTCATTAACTGCAAATGCCCAAACTgctagc | *alr2269*  (mutation in start codon) |

*Nostoc* sequences are capitalized, with black letters corresponding to 5’UTR and green letters corresponding to coding sequences, respectively. NsiI and NheI sites that were used for cloning are highlighted in magenta and yellow, respectively. Nucleotide changes with respect to wild type sequences are indicated in red.
