# Supplementary material for: The Integrity of the Cell Wall and Its Remodeling during Heterocyst Differentiation Are Regulated by Phylogenetically Conserved Small RNA Yfr1 in Nostoc sp. Strain PCC 7120
Source: mBio. 2020 Jan 21;11(1):e02599-19. doi: 10.1128/mBio.02599-19 (PMC6974561; doi:10.1128/mBio.02599-19)
Supplement: TABLE S6 [file mBio.02599-19-st006.docx]

**Table S6.** Sequences of inserts in plasmids used for expression of Yfr1 and its mutated versions*.*

| **Plasmid** | **Sequence** | **Description** |
| --- | --- | --- |
| pMBA1 | AGCGGAGACGCATGTTTCCGTTCACTCCTCACACCACACTCCGCCCGGACTACTGTTCGGGCGGTTCCTTATGTATTACATAAATTCTTTAGCTTCTTTGCAAATGTATCTAGA | Yfr1 |
| pMBA13 | AGCGGAGACGCATGTTTCCGTTCACTCCTCTGACCACACTCCGCCCGGACTACTGTTCGGGCGGTTCCTTATGTATTACATAAATTCTTTAGCTTCTTTGCAAATGTATCTAGA | Yfr1_UG |
| pMBA15 | AGCGGAGACGCATGTTTCCGTTCACTAAAAACACCACACTCCGCCCGGACTACTGTTCGGGCGGTTCCTTATGTATTACATAAATTCTTTAGCTTCTTTGCAAATGTATCTAGA | Yfr1_AAAA |

Grey shadowed letters indicate the Yfr1 sequence. Modified nucleotides are marked in red. XbaI restriction site used for cloning is shown in blue.
